# Massachusetts flu vaccination and application for COVID-19 routine vaccination planning

**DOI:** 10.1016/j.jvacx.2022.100229

**Published:** 2022-10-19

**Authors:** Megan Hatch, R. Monina Klevens

**Affiliations:** Massachusetts Department of Public Health, Boston, MA, United States

**Keywords:** SARS-CoV-2, COVID-19 vaccine, Racial disparities

## Abstract

**Background:**

SARS-CoV-2 has affected communities of color at disproportionate rates. In particular, Black Americans have higher COVID-19 mortality rates, rooted in health disparities and institutionalized racism. We describe Massachusetts (MA) influenza (flu) vaccination data by race and vaccination location to inform yearly COVID-19 vaccination plans.

**Methods:**

We analyzed self-reported, pooled data from the 2017, 2018, and 2019 Massachusetts Behavioral Risk Factor Surveillance System (MA-BRFSS) of adults. Using the questions around race and ethnicity and flu vaccination, we calculated location types most visited as a percent of people receiving flu vaccine, stratified by race.

**Results:**

The 3 years combined yielded 6031 completed surveys. Compared to White non-Hispanics, Black non-Hispanics, Hispanics, and other non-White adults combined reported flu vaccination less frequently (p < 0.01). Doctor’s office or a health maintenance organization (HMO) were the primary flu vaccination locations among all race subcategories. Within each race category, the top three locations covered 82.7 % of White respondents, while covering only 75.9 % of Hispanic respondents, and 71.0 % of Black respondents. Fewer Hispanic (16.1 %) and Black respondents (13.8 %) were vaccinated at supermarkets or drug stores compared to White respondents (25.2 %).

**Conclusion:**

As COVID-19 vaccination will likely be a yearly occurrence, the above findings can help support future COVID-19 vaccination plans. Since the frequency and location of receipt of flu vaccination varied by race/ethnicity in Massachusetts, the state should ensure specific COVID-19 vaccination locations are available going forward.

## 1 Introduction

Over ninety-six million Americans have contracted SARS-CoV-2 since the pandemic started in 2020 [1]. Infected people may suffer long-term, sometimes serious medical complications, including death [2], [3]. Effective vaccines have been developed in record time [4] and are an important component of the US strategy to control transmission and reduce cases and complications. States created preliminary vaccination plans in October 2020 [5], and will need to revise for future years. Many state plans listed healthcare workers, people over 65, and those with severe medical conditions as groups to be vaccinated early [4]. The disproportionate impact of infection on communities of color [6] was highlighted in a report released by the National Academies of Sciences, Engineering, and Medicine, which urged states to reach those communities with vaccination [7]. As of October 2022, just over two hundred and twenty-five million Americans have been fully vaccinated [8], but despite these advancements, communities of color remain the hardest hit, and in some states, disproportionately unvaccinated, particularly Black Americans [9].

Vaccine hesitancy varies by race and ethnicity in the US [10], [11], with an April 2020 study finding willingness in 47 % of Black respondents to receive a COVID-19 vaccine, as opposed to 79 % of White respondents [12]. Although the discrepancy in vaccine hesitancy has been closing the longer the pandemic continues [13], an April 2022 tally reported that across 38 states, Black Americans were less likely than White Americans to have received at least one vaccine dose (57 % vs 63 %) [9]. Safety concerns and distrust of the American healthcare system likely contributed to the lower percentage of Black people surveyed reporting willingness to receive a COVID-19 vaccine [9], [14]. Health disparities are multifactorial, including socio-economic privilege and decades of systemic racism [14], resulting in lower rates of health insurance coverage and barriers to care [15], [16], [17]. One such barrier to care is location of healthcare services [17], which was an early issue in COVID-19 vaccine distribution [18].

As states implement COVID-19 vaccination plans for future years, examining behaviors of Massachusetts residents with other vaccinations may be useful. Prior research found that receipt of flu vaccine was a predictor of willingness to receive a COVID-19 vaccine [19], [20]. The objective of this brief report was to describe self-reported flu vaccination demographics and location of vaccine receipt to inform yearly COVID-19 vaccination plans.

## 2 Methods

Data were from the 2017, 2018, and 2019 Massachusetts Behavioral Risk Factor Surveillance System (MA-BRFSS) surveys combined. Briefly, the BRFSS is an anonymous, cross-sectional, state-based multi-stage probability sample of random-digit–dialed telephone (land lines and cell phones) survey (details here https://www.cdc.gov/brfss/index.html). The BRFSS collects information on perceived health status as well as many of the behaviors and conditions that place adults (aged ≥ 18 years) at risk for chronic diseases. Data on flu vaccine participation was also collected.

The BRFSS asked whether the respondent had received a flu vaccination in the past 12 months, and if so, in which month and year. Specifically, the following question was used to categorize flu vaccination status: “During the past 12 months, have you had either a seasonal flu shot or a seasonal flu vaccine that was sprayed in your nose?” Respondents were also asked to identify the type of location in which they received the vaccination. Race and ethnicity categories were White non-Hispanic, Black non-Hispanic, Hispanic, American Indian or Alaska Native non-Hispanic, Asian non-Hispanic, or Pacific Islander non-Hispanic. Due to the low frequency of individuals (<300) who selected American Indian or Alaska Native non-Hispanic, Asian non-Hispanic, or Pacific Islander non-Hispanic as their race/ethnicity, *and* had completed vaccination location information, for this analysis they were assigned an integrated category (“All other combined”).

The estimates in this report are based on 6031 completed surveys from the combined 2017, 2018, and 2019 survey responses. Differences were assessed using pairwise T-tests and chi-square tests, with p < 0.05 considered statistically significant. The survey was considered to be non-human subjects research by the Massachusetts IRB.

## 3 Results

Overall, 42.3 % of respondents reported receiving a flu vaccine in the previous 12 months, with a statistically significant higher number of women reporting vaccine receipt than men (45.7 % vs 38.7 %, p < 0.0001, Table 1). The frequency of respondents who reported receiving a flu vaccination was higher among White non-Hispanics (43.3 %) followed by 40.4 % of respondents who were Hispanic, 40.0 % of respondents included among the “All other combined” race category, and 37.0 % of respondents who were Black non-Hispanic (Table 1). Compared to White non-Hispanics, the combined percentage of Black non-Hispanic, Hispanic, and other non-White adults reporting flu vaccination was statistically significantly lower (p = 0.0075, Table 1). Age-adjusted prevalence was not statistically significantly different for any race or ethnicity.

**Table 1:** Statistical Testing of Percent of MA Adults By Self- Reported Receipt of Influenza Vaccination in the Previous Year, 2017–2019.

Total n = 12686	n	%	95 % CL		% No vaccine reported	p-value
*Sex*						
Male	5786	38.7	37.0	40.3	61.3	Reference
Female	6900	45.7	44.0	47.3	54.3	<0.0001
Total n = 12426						

*Race/Ethnicity*						
White, NH	10,107	43.4	42.1	44.7	56.6	Reference
Black, NH	716	37.0	32.3	41.7	63.0	0.0096
Hispanic	958	40.4	36.4	44.5	59.6	0.1692
All other combined, NH	645	40.0	34.9	45.0	60.0	0.1956

*Race/Ethnicity, T-test Pairwise comparison*						
White, NH	10,107	43.4	42.1	44.7	56.6	Reference
Non-white and Hispanic	2319	39.3	36.7	42.0	60.7	0.0075

*Race/Ethnicity, age-adjusted prevalence*						
White, NH	Not available	40.8	39.4	42.3	59.2	Reference
Black, NH		36.9	32.2	41.6	63.1	0.1659
Hispanic		43.4	39.3	47.4	56.6	0.4504
All other combined, NH		43.7	38.3	49.0	56.3	0.5172

The location where respondents obtained their flu vaccinations differed slightly by race/ethnicity (Table 2). The top three locations for all respondents combined were a doctor’s office or health maintenance organization (42.5 %), a supermarket or drugstore (22.7 %), or their workplace (13.6 %). These locations covered 82.7 % of White respondents, 75.9 % of Hispanic respondents, 74.9 % of all other combined respondents, and 71.0 % of Black respondents. When looking within race categories, the top three locations where White non-Hispanic respondents received their vaccination were a doctor’s office or health maintenance organization (43.5 %), a supermarket or drugstore (25.2 %), or their workplace (14.1 %), while Hispanic respondents received flu vaccination primarily from a doctor’s office or health maintenance organization (34.3 %), another type of clinic or health center (a community health center) (25.5 %), or a supermarket or drugstore (16.1 %). Other respondents combined received their vaccination from a doctor’s office or health maintenance organization (41.7 %), a supermarket or drugstore (17.0 %), or their workplace (16.2 %), and Black non-Hispanic respondents received their vaccinations at a doctor’s office or health maintenance organization (40.5 %), their workplace (16.6 %), or a supermarket or drugstore (13.8 %).

**Table 2:** Location Where Adults Self-Reported Receipt of Influenza Vaccine by Race/Ethnicity, Massachusetts, 2017–2019.

Total n = 6031	Overall		White, NH		Black, NH		Hispanic		All other combined, NH	
	n	%	n	%	n	%	n	%	n	%
A doctor’s office or health maintenance organization (HMO)	2607	42.5	2171	43.5	129	40.5	147	34.4	100	41.7
A health department	100	1.8	69	1.3	12	5.9	13	3.9	3	0.6
Another type of clinic or health center (a community health center)	376	7.2	238	4.9	25	7.1	83	25.5	24	7.7
A senior, recreation, or community center	83	1.0	75	1.1	2	0.4	2	0.5	4	0.5
A store (supermarket, drug store)	1514	22.7	1343	25.2	46	13.8	59	16.1	44	17.0
A hospital (inpatient or outpatient)	370	6.2	269	5.2	31	9.7	37	9.4	23	9.6
An emergency room	8	0.2	6	0.2	0	.	2	0.3	0	.
Workplace	716	13.6	601	14.1	40	16.6	25	5.4	39	16.2
Some other kind of place	186	3.1	145	3.0	12	5.4	12	3.1	11	3.0
Received vaccine in Canada or Mexico	2	0.0	2	0.0	0	.	0	.	0	.
A school	40	1.2	30	1.2	1	0.4	1	0.6	8	2.8
Don’t know/Not sure	21	0.3	14	0.3	0	.	2	0.4	4	0.9
Refused	8	0.1	5	0.1	0	.	1	0.7	0	.

## 4 Discussion

Fewer than half of MA adults reported receipt of a flu vaccine in 2017–2019, thus a full understanding of the challenges and barriers to higher vaccination coverage is needed. For the primary COVID-19 vaccine roll out, Massachusetts focused on hospitals/healthcare systems, mass vaccination sites, pharmacies, and local boards of health [21] as distribution sites. When we compare this approach to prior flu location for vaccination, several lessons can be learned from flu data. Importantly, ‘one size does not fit all’, and availability of vaccination at a variety of location types will continue to be warranted. While the primary place for flu vaccination was a doctor’s office or healthcare maintenance organization for individuals from all race and ethnic groups, the second most frequent vaccination location varied. The top two places where Black respondents received flu vaccine were doctor’s offices and workplaces (Table 2), but one or both of those sites might not be as available for COVID-19 vaccination. As millions of people have lost their jobs [22] across the US during the pandemic, states should explore how else to reach individuals who normally would get vaccinated at work. Furthermore, although COVID vaccines are currently free to all Americans it is unclear whether this will continue for future years; a change to this would severely impact uninsured populations. While Massachusetts does have near- universal health insurance coverage, gaps still exist among non-elderly adults [23].

Because doctor’s offices were the highest visited place for all respondents, states could maximize vaccine availability at doctor’s offices, for all, not just those with a primary care physician at that location. Information about location and availability of COVID-19 vaccines including for uninsured people should be clearly and widely communicated. Still, because of the multiple barriers people of color face to accessing healthcare, including lack of trust (created by systemic racism) [9], and implicit bias of healthcare providers [24], [25] there is a need to consider these factors to successfully implement COVID-19 vaccination.

The dispersion of where people receive vaccinations will be important to keep in mind as well. This means while 82.7 % of White people can be reached by just three location types, locations *other* than workplaces, doctor’s offices, and stores need to be included to ensure all individuals have access to vaccine (Table 2). Locations to receive vaccine should include places such as clinics, health centers, or other places trusted by communities of color. Community health centers could be particularly important for Hispanic survey respondents, 25.5 % of whom reported going there for flu vaccines (Table 2). Other states have also noted vaccine receipt disparities [26], and have adopted strategies such as prioritizing vaccine allocation to providers who serve disproportionately impacted communities, and investing in trusted community leaders [27]. Washington state saw success with a multitude of these strategies (5 % of Hispanic population vaccinated, raised to 64 % by April 2022) [28]. We recommend building community partnerships and engaging with local trusted community leaders in communications efforts, to learn how to increase both accessibility, and comfort levels for people seeking a vaccine. Transparency and providing answers to frequently asked questions should also be included.

When comparing our results to prior studies, it is important to note that while favored locations may have shifted, disparities in vaccination remain [29], [30], and one study found the disparities have widened with COVID-19 uptake, more than flu ever recorded [31].

A strength of this study was that BRFSS uses a representative sample of the noninstitutionalized adult Massachusetts population and that race/ethnicity is self-reported as perceived. However, limitations include that data are self-reported and subject to respondent and recall bias. Individuals who participate may be more health aware and have healthier behaviors, including vaccination. Additionally, COVID-19 vaccination rates in MA surpasses that of flu [32], meaning there may be patterns we were not able to discern by looking at flu data alone. Lastly, these numbers are specific to Massachusetts, and may not be generalizable to other areas.

## 5 Conclusion

In summary, we found that the receipt of flu vaccine and the location where respondents received vaccine varied by race and ethnicity. Ensuring COVID-19 vaccine is available in a variety of location types, and addressing other barriers to care, might improve coverage among all residents.

## Declaration of Competing Interest

The authors declare that they have no known competing financial interests or personal relationships that could have appeared to influence the work reported in this paper.

## Data Availability

Data will be made available on request.

## References

[1] CDC. COVID-19 cases, deaths, and trends in the US; 2020. <https://covid.cdc.gov/covid-data-tracker/> [accessed 31 May, 2022].

[2] del Rio C., Collins L.F., Malani P. (2020). Long-term health consequences of COVID-19. JAMA.

[3] No authors listed (2020). Understanding the long-term health effects of COVID-19. EClinicalMedicine.

[4] NIH. Statement from NIH and BARDA on the FDA emergency use authorization of the moderna COVID-19 vaccine; 2020. <https://www.nih.gov/news-events/news-releases/statement-nih-barda-fda-emergency-use-authorization-moderna-covid-19-vaccine> [accessed 21 December 2020].

[5] CDC. COVID-19 vaccination program operational guidance; 2020. <https://www.cdc.gov/vaccines/covid-19/covid19-vaccination-guidance.html> [accessed 7 December 2020].

[6] Louis-Jean J., Cenat K., Njoku C.V., Angelo J., Sanon D. (2020). Coronavirus (COVID-19) and racial disparities: a perspective analysis. J Racial Ethn Health Disparities.

[7] National Academies. National Academies Release Framework for Equitable Allocation of a COVID-19 Vaccine for Adoption by HHS, State, Tribal, Local, and Territorial Authorities; 2020. <https://www.nationalacademies.org/news/2020/10/national-academies-release-framework-for-equitable-allocation-of-a-covid-19-vaccine-for-adoption-by-hhs-state-tribal-local-and-territorial-authorities> [accessed 7 December 2020].

[8] CDC. COVID-19 Vaccinations in the United States; 2020. <https://covid.cdc.gov/covid-data-tracker/#vaccinations> [accessed 31 May 2022].

[9] Kaiser Family Foundation. Latest Data on COVID-19 Vaccinations Race/Ethnicity; 2021. <https://www.kff.org/coronavirus-covid-19/issue-brief/latest-data-on-covid-19-vaccinations-race-ethnicity/> [accessed 31 May 2022 ].

[10] Nguyen L.H., Joshi A.D., Drew D.A. (2022). Self-reported COVID-19 vaccine hesitancy and uptake among participants from different racial and ethnic groups in the United States and United Kingdom. Nat Commun.

[11] Bagasra A.B., Doan S., Allen C.T. (2021). Racial differences in institutional trust and COVID-19 vaccine hesitancy and refusal. BMC Public Health.

[12] Kelly BJ, Southwell BG, McCormack LA, et al. Predictors of willingness to get a COVID-19 vaccine in the U.S. BMC Infect Dis. 2021;21;e338. <https://doi.org/10.1186/s12879-021-06023-9>. 10.1186/s12879-021-06023-9 PMC8039496 33845781

[13] Padamsee T.J., Bond R.M., Dixon G.N. (2022). Changes in COVID-19 vaccine hesitancy among black and white individuals in the US. JAMA Netw Open.

[14] Agarwal R, Dugas M, Ramaprasad J, et al. Socioeconomic privilege and political ideology are associated with racial disparity in COVID-19 vaccination. Proc Natl Acad Sci. 2021;118 (33);e2107873118. <https://doi.org/10.1073/pnas.2107873118>. 10.1073/pnas.2107873118 PMC8379950 34326130

[15] Phelan J.C., Link B.G., Tehranifar P. (2010). Social conditions as fundamental causes of health inequalities: theory, evidence, and policy implications. J Health Soc Behav.

[16] Link B.G., Phelan J. (1995). Social conditions as fundamental causes of disease. J Health Soc Behav.

[17] Kaiser Family Foundation. The undefeated survey on race and health - main findings; 2020. <https://www.kff.org/report-section/kff-the-undefeated-survey-on-race-and-health-main-findings/> [accessed 7 December 2020].

[18] Williams, N, Tutrow H, et al. Assessment of Racial and Ethnic Disparities in Access to COVID-19 Vaccination Sites in Brooklyn, NY*.* JAMA Netw Open 2021;4(6); e2113937. doi:10.1001/jamanetworkopen. 2021.13937. 10.1001/jamanetworkopen.2021.13937 PMC8214153 34143195

[19] Jamieson KH, Romer D, Jamieson PE, et al. The role of non-COVID-specific and COVID-specific factors in predicting a shift in willingness to vaccinate: a panel study. Proc Natl Acad Sci USA 2021;118(52):e2112266118. <https://doi.org/10.1073/pnas.2112266118>. 10.1073/pnas.2112266118 PMC8719857 34930844

[20] Ruiz JB, Bell RA. Predictors of intention to vaccinate against COVID-19: Results of a nationwide survey. Vaccine 2021; 39(7); 1080–1086. <https://doi.org/10.1016%2Fj.vaccine.2021.01.010>. 10.1016/j.vaccine.2021.01.010 PMC7794597 33461833

[21] WCVB. Smaller Mass. Clinics Set to Take Bigger Role in COVID-19 Vaccine Effort; 2021. <www.wcvb.com/article/smaller-massachusetts-clinics-set-to-take-bigger-role-in-covid-19-vaccine-effort/36333879#> [accessed 10 June 2021].

[22] U.S. Bureau of Labor Statistics. 6.2 million unable to work because employer closed or lost business due to the pandemic, June 2021; 2021. <https://www.bls.gov/opub/ted/2021/6-2-million-unable-to-work-because-employer-closed-or-lost-business-due-to-the-pandemic-june-2021.htm> [accessed 3 June 2022].

[23] AJMC. Massachusetts Healthcare Reform, 10 Years Later; 2016. <https://www.ajmc.com/view/massachusetts-healthcare-reform-10-years-later> [accessed 14 December 2020].

[24] Chapman E.N., Kaatz A., Carnes M. (2013). Physicians and implicit bias: how doctors may unwittingly perpetuate health care disparities. J Gen Int Med.

[25] FitzGerald C., Hurst S. (2017). Implicit bias in healthcare professionals: a systematic review. BMC Med Ethics.

[26] Kaiser Family Foundation. How are states addressing racial equity in COVID-19 vaccine efforts?; 2021. <https://www.kff.org/racial-equity-and-health-policy/issue-brief/how-are-states-addressing-racial-equity-in-covid-19-vaccine-efforts/> [accessed 14 April 2021].

[27] abcNews. Washington state reveals racial disparities in vaccine rollout; 2021. <https://abcnews.go.com/Health/washington-state-reveals-racial-disparities-vaccine-rollout/story?id=75855048> [accessed 3 June 2022].

[28] Kaiser Family Foundation. Latest Data on COVID-19 Vaccinations by Race/Ethnicity; 2022. <https://www.kff.org/coronavirus-covid-19/issue-brief/latest-data-on-covid-19-vaccinations-by-race-ethnicity/> [accessed 6 June 2022].

[29] Uscher-Pines L., Maurer J., Harris K.M. (2011). Racial and ethnic disparities in uptake and location of vaccination for 2009–H1N1 and seasonal influenza. Am J Public Health.

[30] Vupputuri S., Rubenstein K.B., Derus A.J. (2019). Factors contributing to racial disparities in influenza vaccinations. PLoS ONE.

[31] Watkinson R.E., Williams R., Gillibrand S. (2022). Ethnic inequalities in COVID-19 vaccine uptake and comparison to seasonal influenza vaccine uptake in Greater Manchester, UK: a cohort study. PLoS Med.

[32] USA Facts. How Many COVID-19 vaccines has Massachusetts administered?; 2022. <https://usafacts.org/visualizations/covid-vaccine-tracker-states/state/massachusetts> [accessed 2 June 2022].

